# Controllable NO release from Cu_1.6_S nanoparticle decomposition of *S*-nitrosoglutathiones following photothermal disintegration of polymersomes to elicit cerebral vasodilatory activity†
†Electronic supplementary information (ESI) available: Experimental section and Fig. S1–S10. See DOI: 10.1039/c6sc02774a



**DOI:** 10.1039/c6sc02774a

**Published:** 2016-08-11

**Authors:** Po-Tsung Kao, I-Ju Lee, Ian Liau, Chen-Sheng Yeh

**Affiliations:** a Department of Chemistry and Advanced Optoelectronic Technology Center National Cheng Kung University , Tainan 701 , Taiwan . Email: csyeh@mail.ncku.edu.tw; b Department of Applied National Chiao Tung University , Hsinchu 300 , Taiwan; c Department of Biological Science and Technology National Chiao Tung University , Hsinchu 300 , Taiwan . Email: ianliau@mail.nctu.edu.tw

## Abstract

Since the discovery of nitric oxide (NO) as a vasodilator, numerous NO therapies have been attempted to remedy disorders related to pathological vasoconstriction such as coronary artery disease. Despite the advances, clinical applications of NO therapies remain limited mainly because of the low stability of molecular NO donors (and NO molecules), and concerns about the increased oxidative stress and reduced arterial pressure associated with the systemic administration of NO. Here we design a photo-responsive polymersome with nitrosothiols and Cu_1.6_S nanoparticles in its core and shell, respectively, and demonstrate the photo-triggered release of NO and its vasodilatory activity on zebrafish. Unlike conventional approaches, our design enhances the stability of NO donors and prospectively enables spatiotemporal regulation of NO release, thus minimizing the harmful effects associated with conventional NO therapies. We anticipate that such a strategy will open up new clinical applications of NO and help reveal the complex biological effects of NO *in vivo*.

## Abstract 2

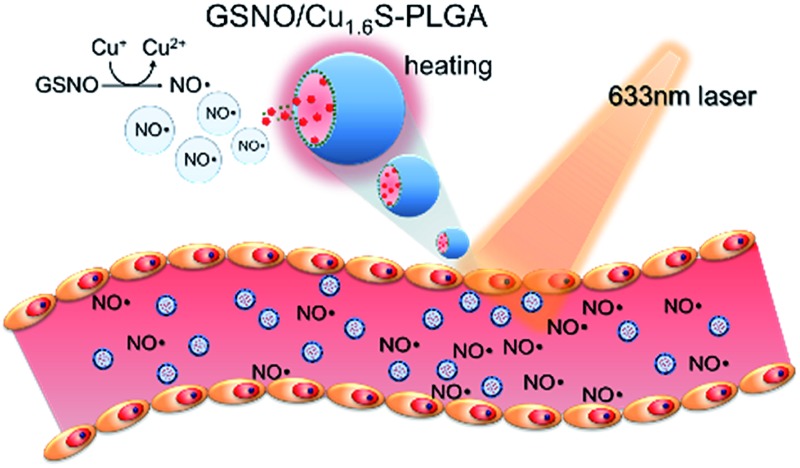
A photo-responsive polymersome was designed to demonstrate the photo-controlled release of NO and its vasodilatory activity on zebrafish.

## Introduction

Since its discovery as a an endothelium-derived relaxing factor,^1^ nitric oxide (NO) has long been recognized as a unique signaling molecule of therapeutic potential. In particular, the uncharged NO molecule, which can be produced either endogenously from vascular endothelium or exogenously from NO donors, readily diffuses into neighboring smooth muscle cells and causes vascular relaxation.^2^ Based on this notion, numerous NO therapies have been developed aiming to improve the blood flow in order to remedy disorders related to pathological vasoconstriction. For instance, nitroglycerin (an NO donor) is administered to patients with coronary artery disease,^3^ and inhalation of NO is suggested to treat pulmonary artery hypertension by reversing hypoxic pulmonary vasoconstriction.^4^ Moreover, a preclinical study of ischemic stroke shows that NO caused selective dilatation of collateral arterioles and resulted in an improved neurological outcome.^5^ Nevertheless, clinical applications of NO therapies remain limited mainly because of concerns over the reduction of arterial pressure and tissue perfusion, and the excessive generation of cytotoxic reactive oxygen/nitrogen species (such as peroxynitrite) after the systemic administration of NO. In addition, the short half-life of NO in biological fluids, which results from its high reactivity to endogenous molecules such as glutathione, albumin hemoglobin and molecular dioxygen, may decrease the bioavailability of NO near the targeted blood vessels.^6–8^ In this regard, development of a strategy to control the generation of NO with high spatiotemporal precision in physiological settings and to increase the bioavailability of NO is hence critically important for optimization of the therapeutic effect of NO. Herein, we present the first example of delivery of NO with controlled release for the direct observation of vasodilation.

**Fig. 1:**
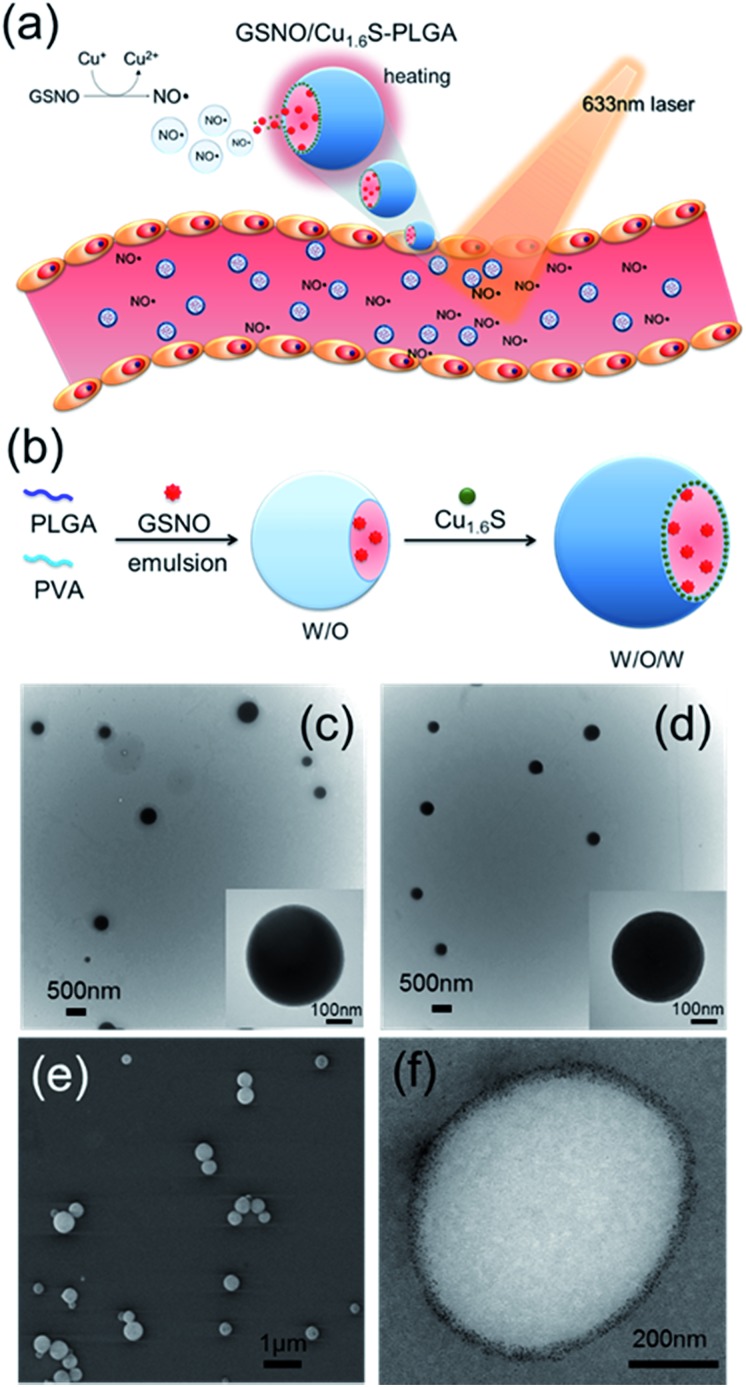
Formulated polymersomes for vasodilation upon laser irradiation, and electron images. (a) Illustration of the GSNO/Cu_1.6_S–PLGA polymersomes applied in vasodilation upon laser irradiation. (b) Preparation of the GSNO/Cu_1.6_S–PLGA polymersomes through a double emulsion process. TEM images of the (c) Cu_1.6_S–PLGA and (d) GSNO/Cu_1.6_S–PLGA polymersomes (inset: magnified image of a single polymersome). (e) SEM image of the GSNO/Cu_1.6_S–PLGA polymersomes. (f) The cryo-TEM image of a GSNO/Cu_1.6_S–PLGA polymersome showing a hollow structure with Cu_1.6_S NPs embedded in the membrane.

Nitrosothiols including *S*-nitrosoglutathione (GSNO), *S*-nitrosoalbumin (AlbSNO) and *S*-nitrosocysteine (CysNO) are found in both human plasma and tissue fluids, and have been suggested to act as a therapeutic NO donor for the treatment of vascular disorders through the anti-platelet, anti-thrombotic and pro-vasodilatory activities of NO.^9,10^ In addition to heat and UV/visible light, which can cause the decomposition of the S–NO bond of nitrosothiols and the subsequent release of NO, metal ions such as Cu^+^, Fe^2+^, Hg^2+^ and Ag^+^ can also react with nitrosothiols leading to the generation of NO.^8,11^ Accordingly, we propose that the djurleite (Cu_2–*x*_S) nanoparticles (NPs) have the potential to take part in the reaction of GSNO for the release of NO. On the other hand, the degradation of polymersomes made of poly(lactic-*co*-glycolic acid) (termed PLGA polymersomes hereafter) is known to be sensitive to heating. Based on these notions, we present a strategy for the photo-triggered generation of NO from a polymersome cargo. Cu_1.6_S NPs have played dual roles in generating heat by laser irradiation and releasing NO by the reaction of GSNO. Our formulation comprises a PLGA polymersome with GSNO encapsulated in the core, and oil-phased djurleite (Cu_2–*x*_S) nanoparticles (NPs) packed inside the membrane, of the polymersome, respectively. Upon irradiation with photons of energy that matches the absorption band of Cu_2–*x*_S NPs, the embedded NPs become heated, thereby causing the increased permeability of the PLGA membrane and the subsequent escape of GSNO molecules from the core of the polymersome. When GSNO molecules infiltrate through the PLGA membrane, they would react with the embedded Cu_2–*x*_S NPs leading to the generation of NO molecules (Fig. 1a). Our design notably improves the stability of NO donors (*i.e.* GSNO) and allows the generation of NO on demand, induced by light. These features are expected to increase the bioavailability of NO and open the possibility to spatiotemporally control the release of NO *in vivo*, based on which novel NO therapies could be envisioned.

## Results and discussion

### Characterization of GSNO/Cu_1.6_S–PLGA polymersomes

Cu_1.6_S NPs were synthesized through a low-temperature (70 °C) redox approach.^12^ TEM images revealed that the Cu_1.6_S NPs were approximately 4.9 nm in size with a *d* spacing of 0.19 nm, which corresponds to the (1 1 3) lattice plane of the crystal (Fig. S1a†). XRD analysis identified particles belonging to the Cu_8_S_5_ phase (JCPDS card no. 33-0491) (Fig. S1b†). Subsequently, the double emulsion method was employed to encapsulate the GSNO molecules and Cu_1.6_S NPs into the hydrophilic core and hydrophobic shells of the PLGA polymersomes, respectively (Fig. 1b). First of all, we prepared Cu_1.6_S–PLGA polymersomes without the inclusion of GSNO. Fig. 1c shows that the corresponding polymersomes can be successfully formed in a spherical morphology with a hydrodynamic diameter of 431 nm. Because GSNO was labile to light and heat, releasing NO,^8,11^ GSNO was added into the hydrophilic core (W/O) under dark and low temperature (4 °C) conditions during the first emulsification process for the preparation of the GSNO/Cu_1.6_S–PLGA polymersomes. Next, the oil-phase Cu_1.6_S NPs were embedded in the membrane of the polymersomes during the second emulsification process. Both TEM and SEM images show that the resultant GSNO/Cu_1.6_S–PLGA polymersomes retained the same morphology as the Cu_1.6_S–PLGA polymersomes but with a slightly increased diameter (441 nm; Fig. 1d and e). As illustrated in a cross-sectional image acquired with cryo-TEM, Cu_1.6_S NPs were distributed mainly in the membrane of the polymersomes (Fig. 1f). However, we can also find that a small portion of Cu_1.6_S NPs were clustered in the cores in some polymersomes (Fig. S2†). The elemental mapping analysis verified the presence of Cu and S in the polymersomes. The line-scan analysis showed a concave profile confirming a hollow structure with Cu and S mainly located around the polymersome membrane (Fig. S3†). An NO assay kit following the Griess test^13^ was employed to determine that 4.12 × 10^–6^ M (4.12 μM) of GSNO was encapsulated from the 200 ppm (copper ion concentration) of GSNO/Cu_1.6_S–PLGA polymersomes. To prevent the particles from being engulfed by macrophages in the blood, the positively charged bovine serum albumin (BSA) engaged in a favorable electrostatic interaction with negatively charged PLGA, thus modifying the surface of the PLGA polymersomes. The amount of BSA adsorbed on the GSNO/Cu_1.6_S–PLGA polymersomes was 13.3 μg BSA in 200 ppm GSNO/Cu_1.6_S–PLGA.

### Evidence of controllable NO release with the reaction of Cu_1.6_S NPs

Prior to performing the laser irradiation of the GSNO/Cu_1.6_S–PLGA polymersomes, the reaction of Cu_1.6_S NPs with GSNO was examined to verify the production of NO. To determine that NO can be generated from the reaction between Cu_1.6_S NPs and GSNO, the oil-phase Cu_1.6_S NPs were transferred to aqueous solution by the modification of hexadecyltrimethylammonium bromide (CTAB) surfactant yielding CTAB–Cu_1.6_S NPs. A solution of CTAB–Cu_1.6_S NPs with a concentration fixed at 100 ppm (copper ion concentration) reacted with a solution of GSNO of varied concentration, and the profile of NO release was determined. The generation of NO increased with the concentration of GSNO, with the NO becoming detectable when the concentration of GSNO exceeded 10^–5^ M (Fig. S4a†). A GSNO solution of fixed concentration (10^–3^ M) was treated with CTAB–Cu_1.6_S NPs solution of varied concentration, and the results show that the release of NO increased with the concentration of CTAB–Cu_1.6_S NPs and reached a plateau at a concentration of 100 ppm (Fig. S4b†); the strong dependence of NO release on the concentration of CTAB–Cu_1.6_S NPs indicates that NO was generated mainly through the reaction between CTAB–Cu_1.6_S NPs and GSNO but not the direct decomposition of GSNO.

**Fig. 2:**
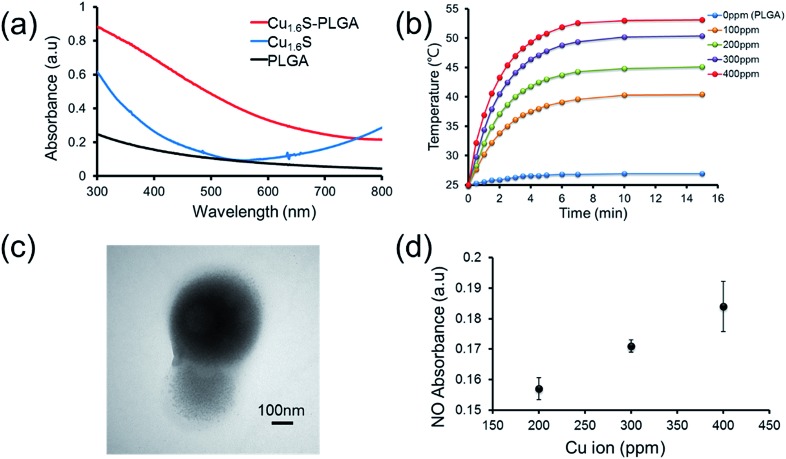
Evidence of NO release upon laser exposure because of hyperthermia-induced polymersome destruction. (a) UV-vis spectra of PLGA, Cu_1.6_S NPs, and Cu_1.6_S–PLGA polymersomes. (b) Temperature elevation profiles of the Cu_1.6_S–PLGA polymersomes irradiated by a 633 nm diode laser at 1 W cm^–2^ for 15 min as a function of polymersome dosage based on Cu ion concentration. (c) TEM image showing the destructed Cu_1.6_S–PLGA polymersome on exposure to a 633 nm diode laser at 1 W cm^–2^ for 15 min. (d) NO release as a function of the GSNO/Cu_1.6_S–PLGA polymersome concentrations on exposure to a 633 nm diode laser at 1 W cm^–2^ for 15 min.

We proceeded to investigate whether NO can be triggered to be released from GSNO/Cu_1.6_S–PLGA polymersomes by light irradiation. The UV-visible absorption spectra of PLGA, Cu_1.6_S NPs and Cu_1.6_S–PLGA polymersomes were analyzed (Fig. 2a). PLGA alone did not have discernible absorption with red light, whereas the absorbance of the Cu_1.6_S NPs began to rise from approximately 600 nm. When the Cu_1.6_S NPs were encapsulated in polymersomes (Cu_1.6_S–PLGA), the absorption increased significantly, which was possibly caused by the aggregation of Cu_1.6_S NPs in the polymersome membrane. The appreciable absorption of Cu_1.6_S–PLGA polymersomes in the red spectral range enables the induction of a photothermal effect with red light, which presumably exerts less phototoxicity to biological cells relative to blue and UV light, and is preferable considering the prospective biomedical applications. Fig. 2b displays the temperature elevation curves obtained from the solutions of Cu_1.6_S–PLGA polymersomes of varied concentration after illumination with a diode laser (*λ* = 633 nm) at 1 W cm^–2^ for 15 min. The temperature of the solutions of all concentrations increased effectively, with the solutions of greater concentration exhibiting more rapid heating. A TEM image confirmed that light irradiation caused destruction of the Cu_1.6_S–PLGA polymersome and a leak-out of Cu_1.6_S NPs from the polymersome (Fig. 2c). Having demonstrated photothermally induced rupturing of the Cu_1.6_S–PLGA polymersomes through the absorption of Cu_1.6_S NPs, we proceeded to examine whether NO was produced from GSNO/Cu_1.6_S–PLGA polymersomes after light irradiation under the same conditions. The release of NO was evidenced and increased with the concentration of polymersomes (represented with the concentration of copper ions) (Fig. 2d). These collective results indicate that NO was generated through the reaction of GSNO with Cu_1.6_S NPs as a result of the disintegration of PLGA polymersomes induced by a photothermal effect.

It is known that the generation of NO through the reaction of nitrosothiols with Cu^+^ is accompanied with the formation of Cu^2+^.^8,11^ To further verify our preceding notion, X-ray photoelectron spectroscopy (XPS) was employed to determine the oxidation states of the copper ions of Cu_1.6_S NPs before and after exposing GSNO/Cu_1.6_S–PLGA polymersomes to light from a 633 nm laser. The binding energies of Cu^+^ are at 932.6 and 952.5 eV, whereas those of Cu^2+^ are at 933.6 and 954.1 eV.^12^ As shown in the XPS spectra, the Cu^2+^/Cu^+^ ratio, which was 0.31 (2P_3/2_) and 0.29 (2P_1/2_) prior to laser irradiation (Fig. S5a†), increased to 0.76 (2P_3/2_) and 0.43 (2P_1/2_) after 15 min of laser irradiation (Fig. S5b†), and hence supports our preceding deduction.

### Evaluation of GSNO stability in polymersomes

As GSNO is unstable under physiological conditions, it would be most desirable if encapsulation of GSNO in PLGA polymersomes could improve the chemical stability of GSNO. Free GSNO decomposed spontaneously at 37 °C, resulting in a significant release of NO within one hour, and the generation of NO reached a maximum at the third hour in both H_2_O and PBS (Fig. S6a†). Although the decomposition of GSNO in polymersomes was still evident, the encapsulation significantly improved the stability of GSNO in both H_2_O and PBS; specifically, the release of NO from GSNO/Cu_1.6_S–PLGA polymersomes in H_2_O was reduced in the time course between 3 and 4 h relative to that from free GSNO (Fig. S6b–e†). Overall, these results show that the encapsulation of GSNO in PLGA polymersomes significantly improved the stability of GSNO relative to free GSNO especially in short runs (*e.g.* <3 h), a favorable feature considering its prospective application as an NO donor under physiological conditions. Another test shows that GSNO encapsulated polymersomes can be effectively stored without disintegration at 4 °C in H_2_O and PBS (Fig. S7†). The low temperature significantly extended the stability of GSNO and prevented the spontaneous release of NO from GSNO for up to 3 days. The SEM images show that most of the GSNO/Cu_1.6_S–PLGA polymersomes retained their general spherical morphology in both H_2_O and PBS at 4 °C after 7 days (Fig. S8†). Careful examination of polymersomes stored in PBS revealed that approximately 13% of the polymersomes showed features of minor to moderate destruction in their structures.

### Photo-triggered intracellular release of NO from polymersomes

**Fig. 3:**
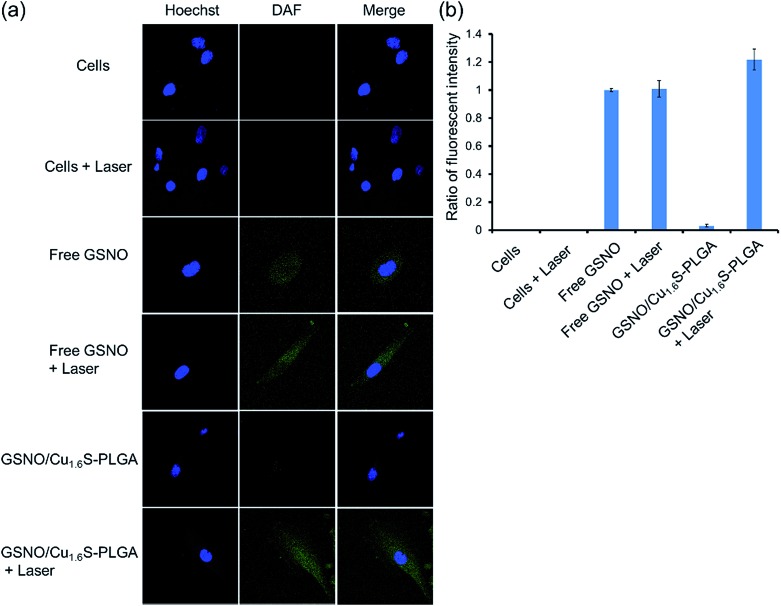
Evidence of intracellular NO release upon laser irradiation under 1 h incubation. (a) Confocal images of the MRC-5 cells captured under different conditions for cells alone, free GSNO (4.12 μM), and GSNO/Cu_1.6_S–PLGA polymersomes (Cu ion concentration 200 ppm with total 4.12 μM encapsulation of GSNO) with or without laser exposure. The nuclei were stained with Hoechst (blue) and the green fluorescence was derived from DAF-2 DA. Laser illumination was performed for 15 min exposure using a 633 nm laser at 1 W cm^–2^. (b) Fluorescence intensities corresponding to the images in (a) were calculated relative to the free GSNO group. The cells were incubated with free GSNO or GSNO/Cu_1.6_S–PLGA polymersomes for 1 h.

We next demonstrated photo-triggered intracellular release of NO on demand *in vitro*. MRC-5 cells were incubated in a medium with or without GSNO/Cu_1.6_S–PLGA polymersomes, and confocal fluorescence images were acquired. The nuclei were highlighted with Hoechst (blue fluorescence), whereas the generation of NO was probed with a fluorescent sensor of NO, DAF-2 DA (green fluorescence) (Fig. 3); when the cell-permeable NO sensor enters cells, it is hydrolyzed to DAF-2 by intracellular esterase, and becomes fluorescent after reaction with NO.^14,15^ For cells alone, no discernible NO-based green fluorescence was seen neither before nor after light irradiation (633 nm, 1 W cm^–2^, 15 min). When cells were cultivated in a medium containing free GSNO (4.12 μM) for 1 h, the green fluorescence became evident, indicating the generation of NO, presumably from the spontaneous decomposition of GSNO at 37 °C; nevertheless, the fluorescence intensity did not vary significantly with or without light irradiation (633 nm, 1 W cm^–2^, 15 min). We proceeded to examine two experimental groups, which corresponded to cells incubated for 1 h in a medium of GSNO/Cu_1.6_S–PLGA polymersomes (200 ppm, corresponding to encapsulated GSNO of 4.12 μM) with or without exposure to light irradiation (633 nm, 1 W cm^–2^, 15 min). No green fluorescence was detected for the group not subjected to light irradiation, a result consistent with our preceding observation that encapsulated GSNO was stable for a few hours. In contrast, strong green fluorescence was observed for the group of cells subjected to light irradiation, and the fluorescence intensity was significantly greater than that observed for cells incubated with free GSNO. Similar results were obtained for the cells cultured in a medium containing GSNO/Cu_1.6_S–PLGA polymersomes (200 ppm) for a longer duration (3 h *vs.* 1 h) (Fig. S9†). Nevertheless, green fluorescence was discernible for the group of cells not subjected to light irradiation; the result indicates that NO was spontaneously generated, which is consistent with the observation of the stability of GSNO–PLGA polymersomes and GSNO/Cu_1.6_S–PLGA polymersomes at 37 °C (Fig. S6†). Comparison of the results obtained for cells incubated with GSNO/Cu_1.6_S–PLGA polymersomes for varied durations (3 h *vs.* 1 h) shows that the longer duration of incubation resulted in a greater fluorescence intensity, which indicates that the uptake of polymersomes by cells increased with the duration of incubation. It is noted that Cu_1.6_S–PLGA polymersomes without encapsulation of GSNO that received a 15 min irradiation by a 633 nm diode laser at 1 W cm^–2^ exhibited at least 90% cell viability, indicating no apparent overheating effect to damage cells.

### Biocompatibility of GSNO/Cu_1.6_S–PLGA polymersomes

**Fig. 4:**
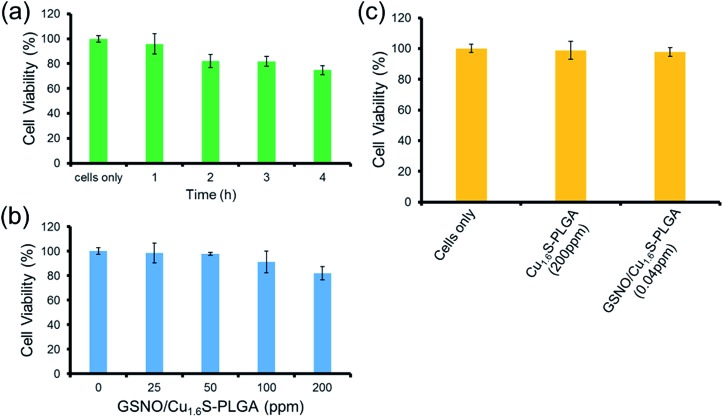
Normal lung fibroblast cells, MRC-5 cells, used as a cell model to evaluate *in vitro* cytotoxicity. (a) Cell viability of MRC-5 cells incubated with 200 ppm (Cu ion concentration) of the GSNO/Cu_1.6_S–PLGA polymersomes with 4.12 μM encapsulation of GSNO in different incubation periods at 37 °C. (b) Cytotoxicity of MRC-5 cells incubated with different dosages (Cu ion concentration) of the GSNO/Cu_1.6_S–PLGA polymersomes for 2 h at 37 °C. (c) Biocompatibility for MRC-5 cells incubated with 200 ppm (Cu ion concentration) of the Cu_1.6_S–PLGA polymersomes and 0.04 ppm (Cu ion concentration used for zebrafish imaging experiments) of the GSNO/Cu_1.6_S–PLGA polymersomes with 0.82 nM encapsulation of GSNO for 24 h at 37 °C.

The toxicity of the GSNO/Cu_1.6_S–PLGA polymersomes was assessed on the cell line of MRC-5 using the MTT assay. The protocols of the preceding fluorescence imaging experiments were followed, except cells were incubated with GSNO/Cu_1.6_S–PLGA polymersomes for varied durations (1, 2, 3 and 4 h) at 37 °C; the survival rate exceeded 95% for an incubation of 1 h but decreased to 80% when the incubation was prolonged to 4 h (Fig. 4a). Cellular survival was also examined for cells incubated with GSNO/Cu_1.6_S–PLGA polymersomes of varied doses but for the same duration (2 h); the viability of the cells decreased slightly with the dose of GSNO/Cu_1.6_S–PLGA polymersomes (Fig. 4b). Particularly, under 2 h incubation, the GSNO/Cu_1.6_S–PLGA polymersomes exerted some toxicity but still had good biocompatibility with cells, with a survival rate of at least 85% up to a dosage of 200 ppm; conversely, Cu_1.6_S–PLGA polymersomes (200 ppm) with no inclusion of GSNO exerted negligible toxicity to cells incubated for 24 h at 37 °C (Fig. 4c). These observations indicate that NO released from GSNO can cause a certain degree of cytotoxicity. In short, these results show that the biocompatibility of GSNO/Cu_1.6_S–PLGA polymersomes can be adjusted through the dose and the duration of incubation. Notably, the dosage used for the later vasodilation experiments in zebrafish (0.04 ppm of GSNO/Cu_1.6_S–PLGA polymersomes with 0.82 nM encapsulation of GSNO) displayed no toxicity in cells after 24 h of incubation at 37 °C (Fig. 4c).

### Cerebral vasodilation in zebrafish model

Having demonstrated the photo-triggered generation of NO *in vitro*, the *in vivo* studies were performed to investigate the possibility of inducing the generation of NO by light and the bioactivity of the generated NO using zebrafish (*Danio rerio*) as a model. Before the experiments, we first validated the protocols for the detection of NO using a fluorescent probe (DAF-FM DA) in living zebrafish, and for the determination of the diameter of cerebral blood vessels of zebrafish. For the latter, a transgenic line of zebrafish, Tg(kdrl:mcherry), which expresses mCherry in the vascular endothelium, was employed. As described, GSNO decomposes spontaneously to produce NO, and hence was chosen to act as an NO donor for this test. As expected, the fluorescence intensity was much stronger for the larva soaked sequentially with GSNO accompanied with the NO probe relative to that soaked with the NO donor alone (Fig. S10a†). On further examination using the transgenic zebrafish, the larva soaked with GSNO exhibited significant vasodilation with the diameter of the basilar artery increasing from 12.9 μm to 15.4 μm; in contrast, the untreated control showed a negligible change in the vascular diameter (Fig. S10b†).

**Fig. 5:**
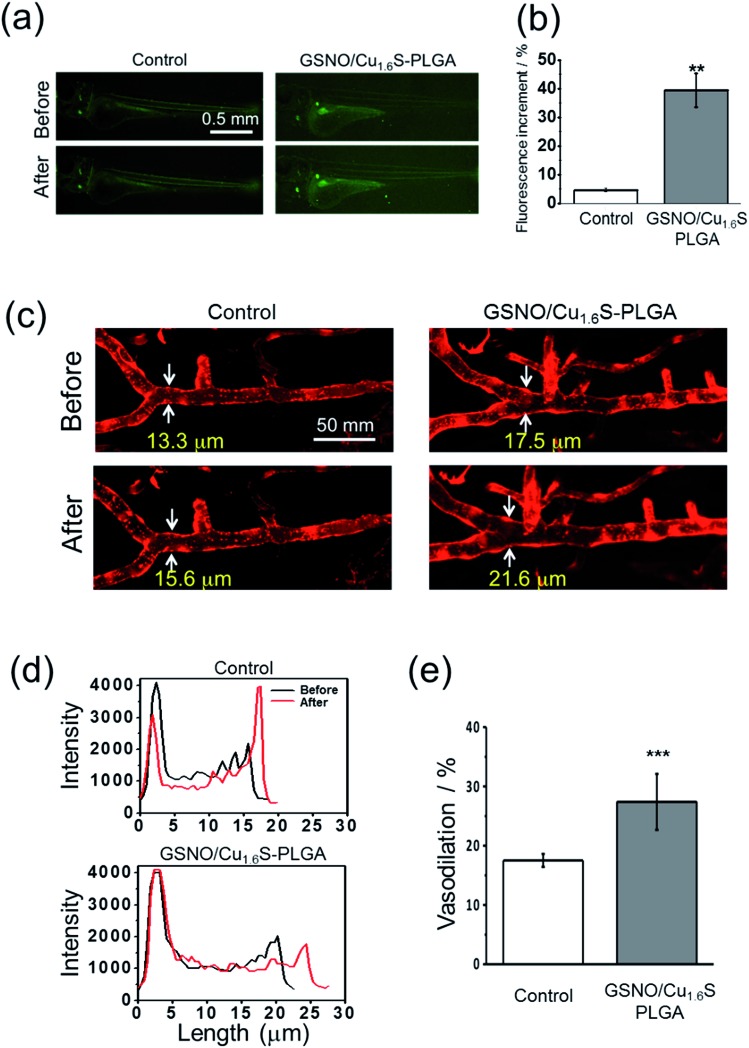
Demonstration of the vasodilatory activity of the photo-induced release of NO from GSNO/Cu_1.6_S–PLGA polymersomes on the cerebral blood vessel of larval zebrafish. (a) Confocal fluorescence images show profoundly increased fluorescence after photo-irradiation (*λ* = 633 nm, 1.6 mW cm^–2^) on zebrafish (wild type, 5 dpf) injected with GSNO/Cu_1.6_S–PLGA polymersomes (200 ppm). (b) Quantitative analysis of the fluorescence images shows that the increase of the fluorescence in zebrafish injected with polymersomes is significantly greater than that of the control (39.4 ± 5.8% *vs.* 4.7 ± 0.6%; *n* = 3, *p* < 0.01). (c) Fluorescence images show a profoundly increased width of the basilar artery after photo-irradiation on zebrafish injected with GSNO/Cu_1.6_S–PLGA polymersomes. All experimental conditions are the same as were used in the preceding experiment except that a transgenic line of zebrafish, Tg(kdrl:mcherry), was employed to highlight the blood vessel. (d) Cross-sectional profiles of the basilar artery of larval zebrafish injected with polymersomes obtained before and after photo-irradiation. (e) Quantitative analysis of the fluorescence images shows that the increase of the width of the basilar artery in the zebrafish injected with polymersomes is significantly greater than that of the control (27.4 ± 4.7%, *n* = 8 *vs.* 17.5 ± 1.1%, *n* = 4; *p* < 0.001).

We then proceeded to examine whether light irradiation can cause NO release *in vivo*. The green fluorescence increased significantly after the irradiation of red light on larvae that were injected with GSNO/Cu_1.6_S–PLGA polymersomes; in contrast, irradiation on the control group, which was not injected with GSNO/Cu_1.6_S–PLGA polymersomes, caused only a minor increase in the fluorescence (Fig. 5a). Statistical analysis shows further that the percentage increase of the fluorescence for the experimental group is significantly greater than that of the control group (39.4 ± 5.8% *vs.* 4.7 ± 0.6%; *n* = 3, *p* < 0.01) (Fig. 5b).

Similar to the preceding test, larvae of transgenic zebrafish, Tg(kdrl:mcherry), were injected with GSNO/Cu_1.6_S–PLGA polymersomes, irradiated by red light, and then imaged with confocal microscopy. Light irradiation caused a dramatic increase in the diameter of the basilar artery; distinctly, light irradiation caused less of an increase in the diameter of the basilar artery of a larva that was injected with a PBS solution containing no polymersomes (Fig. 5c and d). In particular, the percentage increase in the vascular diameter was significantly greater than that observed for the control (27.4 ± 4.7%, *n* = 8 *vs.* 17.5 ± 1.1%, *n* = 4; *p* < 0.001) (Fig. 5e). With these results taken together, we conclude that NO can be triggered by light to release from GSNO/Cu_1.6_S–PLGA polymersomes *in vivo*, and such photo-triggered generation of NO can exert vasodilatory activity by enlarging the diameter of cerebral blood vessels of larval zebrafish.

## Conclusions

In this study, we presented an exogenous stimulus strategy to achieve the stimuli-responsive release of NO on demand. Our study represents for the first time NO produced by stimulus through the reaction between nanomaterials and GSNO and illustrates a prospective application of our design to act as a photo-controllable vasodilator. The release of NO can be controlled by light irradiation when GSNO and Cu_1.6_S NPs are packed in the core and membrane of PLGA polymersomes, respectively. The encapsulation of GSNO in polymersomes also improves the stability of GSNO, which would otherwise decompose spontaneously under physiological conditions. We anticipate that the increased stability of encapsulated GSNO and the unique ability of photo-triggered NO release will open up new possibilities of novel NO based therapies.
