# Supplementary material for: Controllable NO release from Cu1.6S nanoparticle decomposition of S-nitrosoglutathiones following photothermal disintegration of polymersomes to elicit cerebral vasodilatory activity
Source: Chem Sci. 2016 Aug 11;8(1):291–7. doi: 10.1039/c6sc02774a (PMC5365067; doi:10.1039/c6sc02774a)
Supplement: Supplementary file 1 [file SC-008-C6SC02774A-s001.pdf]

Supporting Information

Controllable NO-release from Cu1.6S nanoparticles decomposition of s-nitrosoglutathiones following photothermal disintegration of polymersomes to elicit cerebral vasodilatory activity

Po-Tsung Kao[a], I-Ju Lee[b], Ian Liau[b,c]*, Chen-Sheng Yeh[a]*

a. Department of Chemistry and Advanced Optoelectronic Technology Center, National Cheng Kung University, Tainan, Taiwan.
   E-mail:csyeh@mail.ncku.edu.tw
b. Department of Applied Chemistry, National Chiao Tung University, 1001 University Road, Hsinchu 300, Taiwan
c. Department of Biological Science and Technology, National Chiao Tung University, 1001 University Road, Hsinchu 300, Taiwan
   E-mail: ianliau@mail.nctu.edu.tw

Experimental Section

Materials. All reagents were of analytical purity and used without further purification. Copper(II) acetylacetonate ($Cu(C_5H_7O_2)_2$, 99.9%), sulfur (S, 99.98%), oleylamine ($CH_3(CH_2)_7CH=CH(CH_2)_7CH_2NH_2$, 70%), poly(vinyl alcohol) (PVA, M.W.= 47000, 98%), s-nitrosoglutathione ($C_{10}H_{16}N_4O_7S$, 97%), DAF-2 DA ($C_{24}H_{18}N_2O_7$), albumin from bovine serum (BSA, 98%), para-formaldehyde ($HO(CH_2O)_nH$, 95%), Hoechst 33342 ($C_{27}H_{28}N_6O{\cdot}3HCl{\cdot}3H_2O$, 98%), and 3-(4,5-dime-thylthiazol-2-yl)-2,5-diphenyltetrazolium bromide (MTT, $C_{18}H_{16}BrN_5S$, 97.5%) were used as purchased from Sigma-Aldrich. Poly(lactic-co-glycolic acid) (PLGA, 5050 DLG 4.5A, M.W.= 66 kDa) was bought from Evonik Industries. Chloroform ($CHCl_3$, 99.8%) was obtained from MERCK. Nitric acid (HNO_3, 65%), and hydrochloric acid (HCl, 36%) were required from BASF. Nitric Oxide Colorimetric Assay Kit was purchased from BioVision.

Characterization. Morphology and characterization of the nanoparticles (NPs) and polymersomes were monitored by the transmission electron microscopy (TEM, Hitachi H-7500) and high-resolution transmission electron microscopy (HR-TEM, JEOL JEM-2100F). Surface morphology of the polymersomes was observed by the high-resolution scanning electron microscope (HR-SEM, JEOL JSM-7001F). Concentrations of the NPs were quantified by an inductively coupled plasma-atomic emission spectrometer (ICP-AES, Jobin Yvon JY138 Spectroanalzer). UV−vis spectra were recorded on the UV−vis spectroscopy system (Agilent 8453). X-ray diffraction patterns of NPs were obtained by a X-ray diffractometer (XRD, Shimasz Cu Kα radiation (λ = 1.5418 Å, 30 kV, 30 mA)**.** The nitric oxide colorimetric assay and the quantification of cell viability were performed using an enzyme-linked immune-sorbent assay reader (ELISA reader, Thermo Scientific Multiskan EX). The copper oxidation states were determined by X-ray photoelectron spectroscopy (XPS, Kratos Axis Ultra DLD). Cellular fluorescence images were taken by confocal laser scanning microscope (Nikon inverted research microscope ECLIPSE Ti).

Preparation of $Cu_{1.6}S$ NPs. 64 mg of sulfur was dissolved in 12 mL of oleylamine and stirred for 30 min in oil bath at 70 °C until sulfur totally dissolved in oleylamine. To this solution, we injected a mixture solution containing 20 mL of chloroform, 5 mL of oleylamine, and 533.5 mg of copper(II) acetylacetonate and stirred for another 30 min, and then the color of solution turned dark green. The products were collected by centrifugation (85000 rpm, 10 min) and washed several times using chloroform and

ethanol.

Preparation of $Cu_{1.6}S$-PLGA polymersomes. The preparation of $Cu_{1.6}S$-PLGA was conducted through a double emulsion (water/oil/water) method. The cavity of the polymersome is hydrophilic while the membrane between the core and the outer shell is hydrophobic. Therefore, the oiled phase $Cu_{1.6}S$ NPs can be embed in the hydrophobic membrane. Firstly, we prepared the oil phase solution containing 4 mL of dichloromethane with 10 mg/mL of PLGA. The water phase solution was prepared by 2 mg of PVA dissolved in 200 μL water. For the first emulsion process, we slowly injected the PVA solution into the oil phase PLGA solution by syringe under sonication in ice-water bath condition for 30 min. In the next step, the 8000 ppm of $Cu_{1.6}S$ NPs were dispersed in 500 μL of dichloromethane and mixed with the solution from the first emulsion process. To fill the $Cu_{1.6}S$ into oil phase membranes of the PLGA polymersomes, 12 mL of PVA solution (10mg/mL) was added to the first emulsion solution containing $Cu_{1.6}S$ NPs for second emulsion step using homogenizer in ice-water bath for 20 min to produce $Cu_{1.6}S$-PLGA polymersomes (w/o/w structure). The products were collected by centrifugation (1200 rpm, 2 min) to remove micro-sized polymersomes.

Preparation of GSNO/$Cu_{1.6}S$-PLGA polymersomes. First of all, we prepared the oil phase solution containing 4 mL of dichloromethane with 10 mg/mL of PLGA. The water phase solution was prepared by 2 mg of PVA dissolved in 100 μL of water and mixed with the 100μL of GSNO solution ($2x10^{-3}$M). For the first emulsion process, we slowly injected the PVA and GSNO mixture solution into oil phase PLGA solution by syringe under sonication in ice-water bath condition for 30 min in the dark. Next, the 8000 ppm of $Cu_{1.6}S$ NPs were dispersed in 500 μL of dichloromethane and mixed with the first emulsion solution. To fill the $Cu_{1.6}S$ into oil phase membranes of the PLGA polymersomes, 12 mL of PVA solution (10mg/mL) was added into the first emulsion solution containing $Cu_{1.6}S$ NPs for second emulsion step using homogenizer in ice-water bath for 20 min in the dark to produce GSNO/$Cu_{1.6}S$-PLGA polymersomes (w/o/w structure). The products were processed by centrifugation (1200 rpm, 2 min) to remove micro-sized polymersomes and collected the supernatants for further purification. The supernatants were centrifuged (3500 rpm, 5 min) again to collect the precipitates, GSNO/$Cu_{1.6}S$-PLGA polymersomes, which were washed by water. Finally, 100μL solution containing 60 μg BSA was added into the product solution to conjugate on the polymersomes surface and shaked the solution for 2 h in water-ice bath without light.

Preparation of GSNO-PLGA polymersomes. The oil phase solution containing 4 mL dichloromethane with 10 mg/mL of PLGA was prepared. The water phase solution was prepared by 2 mg of PVA dissolved in 100 μL water and mixed with the 100μL of GSNO solution (2x10^{-3}M). For the first emulsion process, we slowly injected the PVA and GSNO mixture solution into oil phase PLGA solution by syringe under sonication in ice-water bath condition for 30 min in the dark. Next, the first emulsion solution was added into 12 mL of PVA solution (10mg/mL) for second emulsion step using homogenizer in ice-water bath for 20 min in the dark to produce GSNO-PLGA polymersomes (w/o/w structure). The products were processed by centrifugation (1200 rpm, 2 min) to remove micro-sized polymersomes and collect the supernatants for further purification. The supernatants were further centrifuged (3500 rpm, 5 min) to collect the precipitates, GSNO-PLGA polymersomes, which were washed by water. Finally, 100μL solution containing 60 μg BSA was added into the product solution to conjugate on the polymersomes surface and shaked the solution for 2 h in water-ice bath without light.

Temperature elevation profile upon laser irradiation. $Cu_{1.6}S$ NPs and $Cu_{1.6}S$-PLGA polymersomes with different Cu ion concentrations in water were added to the 96-well plates. The solutions irradiated with 633 nm laser for 15 min at 1 W/cm^2 and the laser light with a beam size of 0.21 cm^2. The change of temperature in solutions was determined by a thermocouple coupled with a digital thermometer (TES 1319A-K type).

NO release measurements from the reaction of $Cu_{1.6}S$ NPs with GSNO. The $Cu_{1.6}S$ NPs were firstly modified by CTAB under sonication for 4 h, and then transferred to water phase. 100 ppm $Cu_{1.6}S$ NPs modified with CTAB were dispersed in water to react with various concentrations of GSNO in the dark. In addition, a fixed 10^{-3}M GSNO was used to react with various concentrations of $Cu_{1.6}S$ NP based on copper ion concentrations in the dark. All of the reactions were measured using NO assay kit and ELISA reader.

NO release from GSNO/$Cu_{1.6}S$-PLGA polymersomes upon 633nm laser irradiation. 200, 300, 400 ppm of GSNO/$Cu_{1.6}S$-PLGA polymersomes in water were added to the 96-well plates. The solutions were irradiated with 633 nm laser for 15 min at 1 W/cm^2 with a laser beam size of 0.21 cm^2. After irradiation, NO assay kit was used to measure the amount of NO release.

Stability performance of GSNO and GSNO/$Cu_{1.6}S$-PLGA polymersomes. Pure GSNO dissolved in water for 0, 1, 2, 3, 4, 6, 24 h at 37 °C. The 200 ppm of

$GSNO/Cu_{1.6}S$-PLGA polymersomes were also dispersed in water and PBS (pH 7.4) for 0, 1, 2, 3, 4, 6, 24 h at 37 °C. In addition, the 200 ppm of $GSNO/Cu_{1.6}S$-PLGA polymersomes were dispersed in water and PBS (pH 7.4) for 0, 1, 2, 3, 4 and 7 days, and the solutions were stored at 4 °C. The SEM was used for analysis of polymersomes structures. The NO assay kit measured the amount of NO release by ELISA reader.

Cell culture. MRC-5 cells (human normal lung fibroblast cell line) were cultured in Eagle's Minimum Essential Medium containing 10% FBS in the incubator at 37 °C with 5% CO_2. Cells were generally plated in cell culture dishes and allowed to adhere for 24 h harvested by treatment with 0.25% trypsin solution.

Cytotoxicity studies. In vitro cytotoxicity of free GSNO (4.12 μM) and polymersomes were evaluated by MTT assay of MRC-5 cells. Cells were seeded into 96-well plates with $8x10^3$ cells/well in Eagle's Minimum Essential Medium containing 10% FBS at 37 °C with 5% CO_2 for 24 h. Condition 1: 200 ppm of $GSNO/Cu_{1.6}S$-PLGA polymersomes added into cells with medium and incubated 1, 2, 3, 4 h at 37 °C. Condition 2: $GSNO/Cu_{1.6}S$-PLGA polymersomes with a series of copper ion concentrations were added into cells with medium and incubated for 2 h at 37 °C. Condition 3: 100 μL of free GSNO (4.12 μM), 200 ppm of $Cu_{1.6}S$-PLGA polymersomes, and 0.04 ppm of $GSNO/Cu_{1.6}S$-PLGA polymersomes were added into cells with medium and incubated for 24 h at 37 °C. MTT assays were evaluated to determine cell viability.

Fluorescence detection of NO in MRC-5 cells : MRC-5 cells were seeded into 8 well chamber slides with $7x10^3$ cells/well in Eagle's Minimum Essential Medium containing 10% FBS at 37 °C with 5% CO_2 for 24 h. For the detection purpose, we changed the medium with MEM/FBS containing the $5x10^{-6}$ M DAF-2 DA and incubated at 37 °C for 20 min. The cells washed by PBS solution three times to remove excess probe and added medium for 15 min to complete de-esterification of the intracellular diacetates. The 200 ppm of $GSNO/Cu_{1.6}S$-PLGA polymersomes were added to MRC-5 cells with fresh medium and incubated for 1 and 3 h. Then, we washed by PBS twice to remove the uninternalized polymersomes and replaced with fresh medium. The control groups with cells only incubated for 1 and 3 h. For exposure of laser, the cells were irradiated with 633 nm laser for 15 min at 1 W/cm^2. After treatment, cells were fixed with 4% paraformaldehyde for 20 min at 37 °C. The nuclei were stained with Hoechst 33342. The cells were subjected to observation using a confocal laser scanning microscope.

Determination of the exogenous generation of NO from GSNO in living zebrafish and its vasodilatory activity on the cerebral blood vessel of zebrafish: The zebrafish experiments were performed in compliance with the relevant laws and institutional guidelines and approved by the Animal Investigation Committee of National Chiao Tung University, Taiwan. To determine the generation of NO in zebrafish, larval zebrafish (wild type, 5 dpf) were soaked sequentially in a solution of GSNO (50 μM) for 1 h and then a solution of DAF-FA DA (5 μM) in the dark for 2 h at 28 °C. To determine the vasodialatory activity from the exogenous NO, transgenic zebrafish (Tg (kdrl:mcherry), 5 dpf), which expressed fluorescent mCherry in the vascular endothelium, were soaked in a solution of GSNO (5 μM) for 1 h. The larvae were then washed with E3 medium (NaCl 5 mM, KCl 0.17 mM, $CaCl_2$ 0.4 mM, and $MgSO_4$ 0.16 mM) 3 times, anesthetized with a mixture of tricaine and isoflurane (100 ppm for ech) for 15 min before imaging with a confocal microscope (SP5, Leica TCS SP5 II, Leica).

Demonstration of the vasodilatory activity of photo-induced release of NO from GSNO/$Cu_{1.6}$S-PLGA polymersomes on the cerebral blood vessel of larval zebrafish.: The zebrafish experiments were performed in compliance with the relevant laws and institutional guidelines and approved by the Animal Investigation Committee of National Chiao Tung University, Taiwan. To determine the photo-induced generation of NO from GSNO/$Cu_{1.6}$S-PLGA polymersomes in vivo, larval zebrafish (wild type, 5 dpf) were soaked in a solution of DAF-FA DA (5 μM) in the dark for 2 h at 28 °C, washed with E3 medium (NaCl 5 mM, KCl 0.17 mM, $CaCl_2$ 0.4 mM, and $MgSO_4$ 0.16 mM) 3 times, and then anesthetized with a mixture of tricaine and isoflurane (100 ppm for each) for 15 min. Before imaging, the larvae were injected with a solution containing GSNO/$Cu_{1.6}$S-PLGA polymersomes (200 ppm) and then irradiated with a laser (λ = 633 nm, 1.6 mW/cm^2). To demonstrate the vasodilatory activity of the photo-induced generation of NO from GSNO/$Cu_{1.6}$S-PLGA polymersomes, the transgenic zebrafish (Tg (kdrl:mcherry), 5 dpf) were anesthetized with a mixture of tricaine and isoflurane (100 ppm for each) for 15 min, injected with a solution containing GSNO/$Cu_{1.6}$S-PLGA polymersomes (200 ppm), and then irradiated with a laser (λ = 633 nm, 1.6 mW/cm^2) before imaging with a confocal microscope.

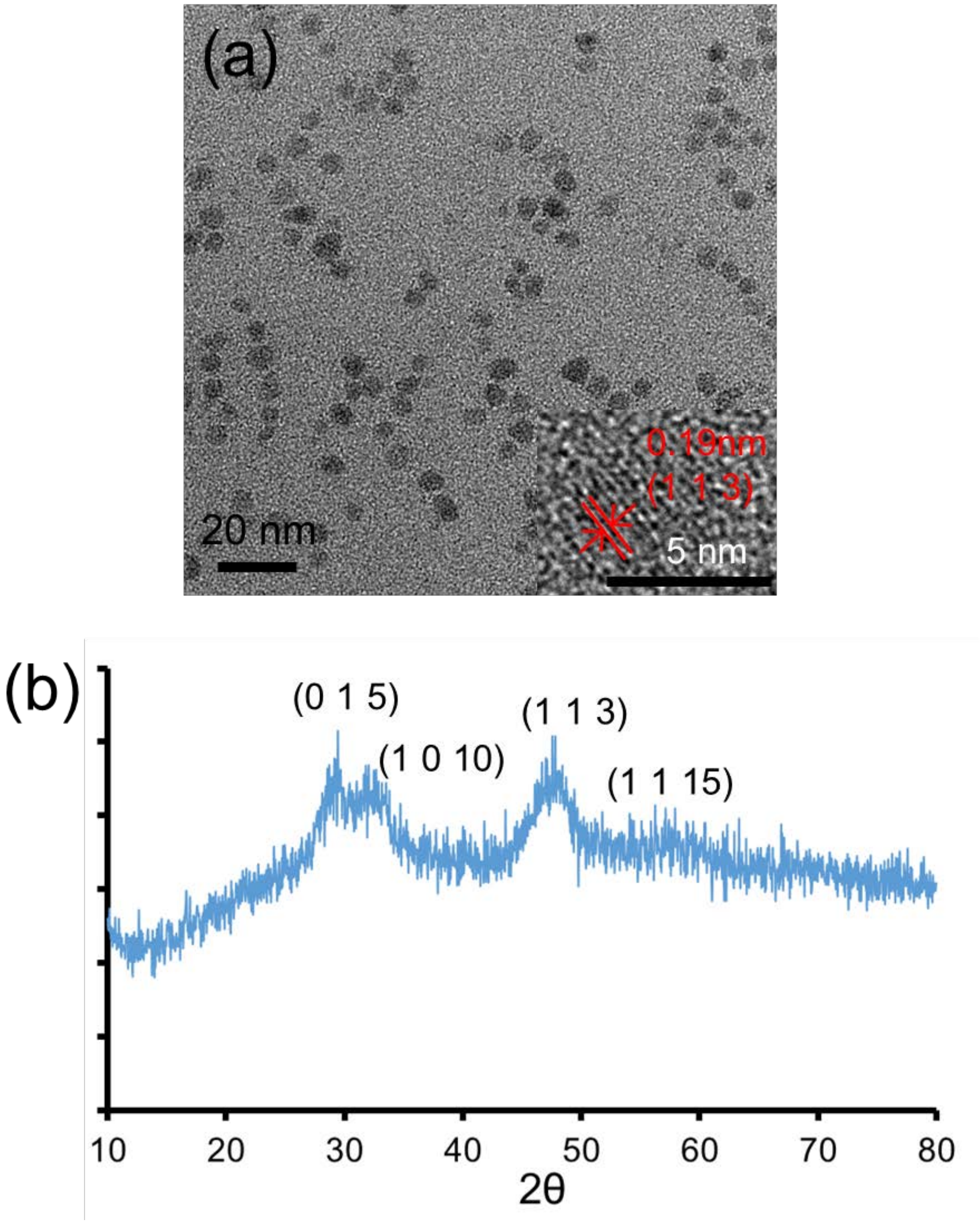

Figure S1. HR-TEM image and XRD spectrum of $Cu_{1.6}S$ NPs. (a) HR-TEM shows 4.9 nm-sized $Cu_{1.6}S$ NPs with (113) crystal plane. (b) XRD identified Cu_8S_5 phase (JCPDS Card No. 33-0491).

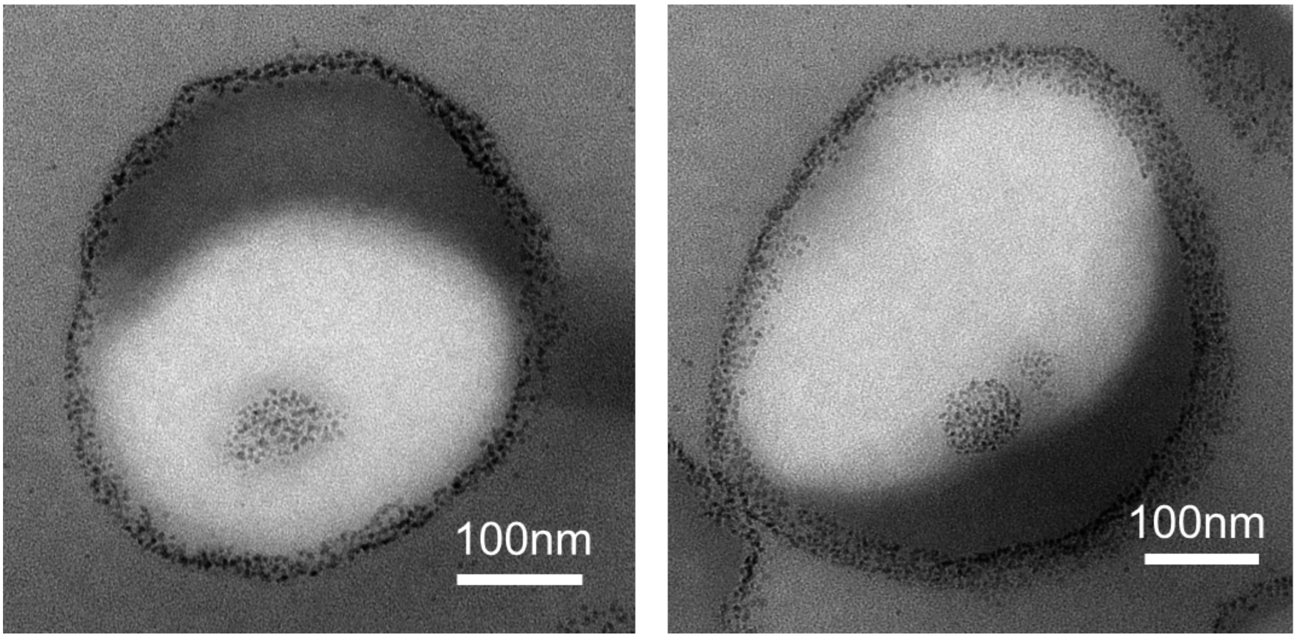

Figure S2. **The cryo-TEM images of the GSNO/$Cu_{1.6}$S-PLGA polymersomes.** Some GSNO/$Cu_{1.6}$S-PLGA polymersomes with $Cu_{1.6}$S NPs clustered in the cores.

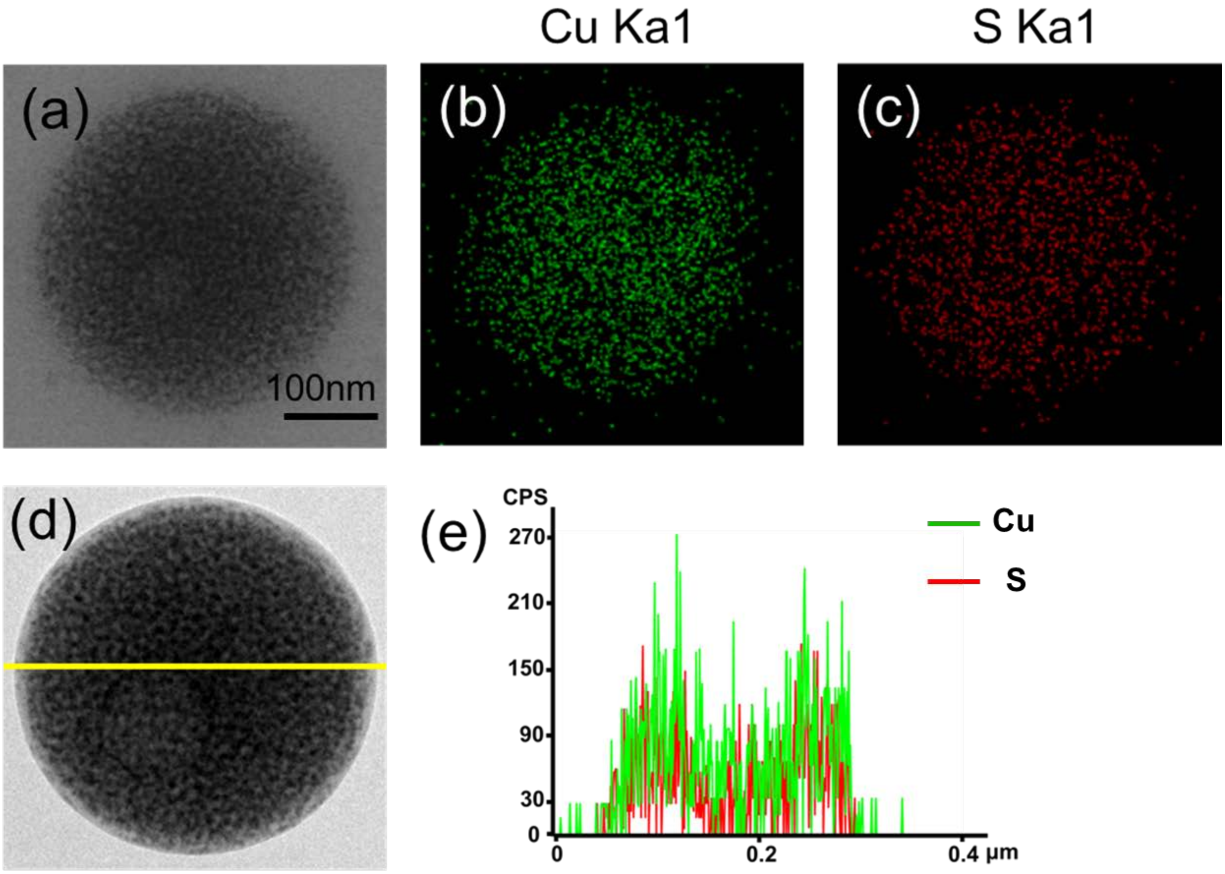

Figure S3. HR-TEM image and EDS analysis of a single GSNO/$Cu_{1.6}$S-PLGA polymersome. The HR-TEM image **(a)** and the corresponding elemental analysis **(b,c)**. **(d)** The EDS line scan profile following the path of electron beam indicated as yellow line. **(e)** The line-scan showing the signal of elements (Cu and S) corresponding to **(d)** distributed in the polymersome membrane. The EDS analysis was captured on the nickel TEM grids.

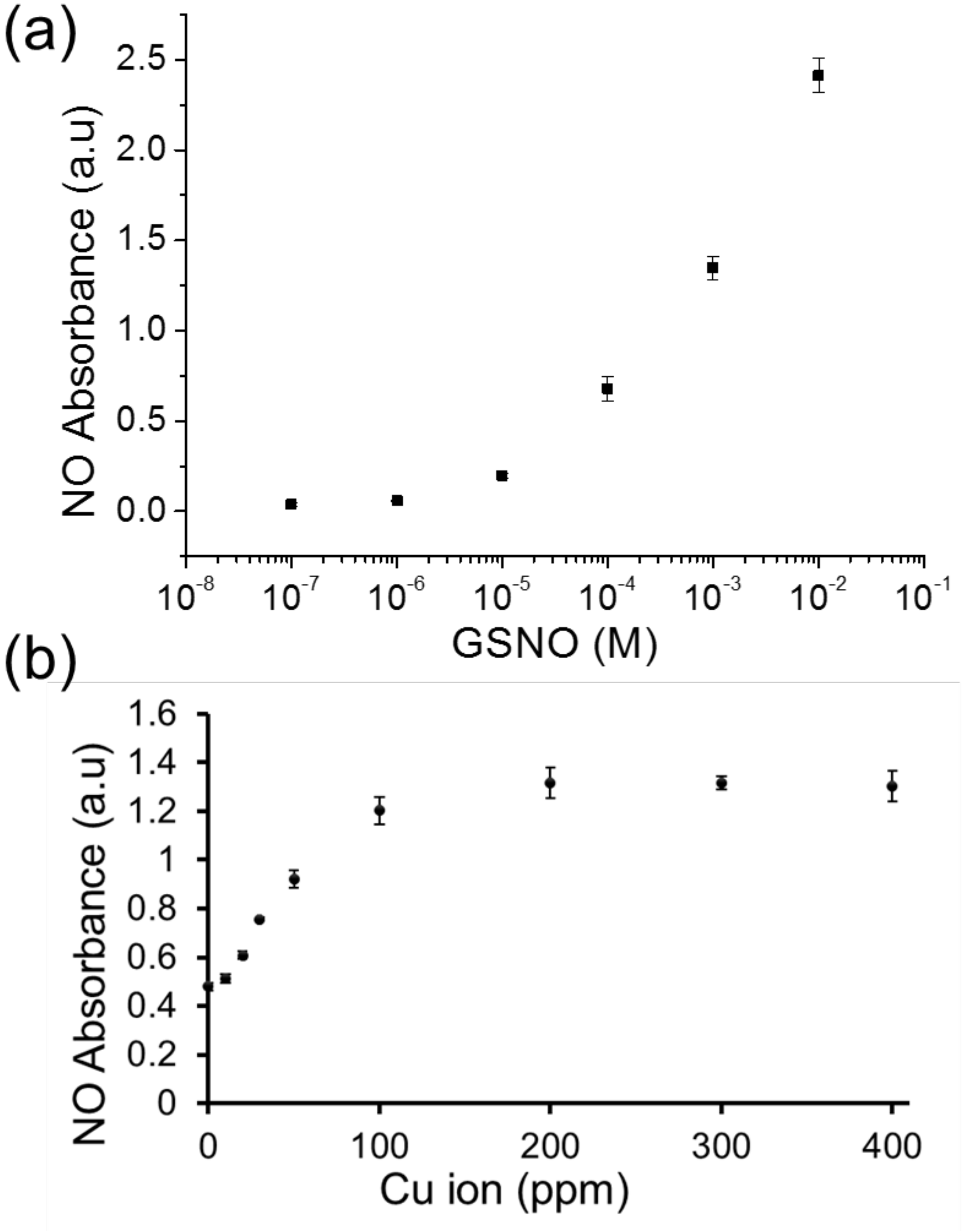

Figure S4. NO detection using nitric oxide colorimetric assay from the reaction of GSNO with CTAB-$Cu_{1.6}S$ NPs. (a) NO release from the reaction of 100 ppm (Cu ion concentration) of CTAB-$Cu_{1.6}S$ NPs as a function of GSNO concentration. **(b)** NO release from the reaction of the fixed 10^{-3}M GSNO as a function of CTAB-$Cu_{1.6}S$ concentration.

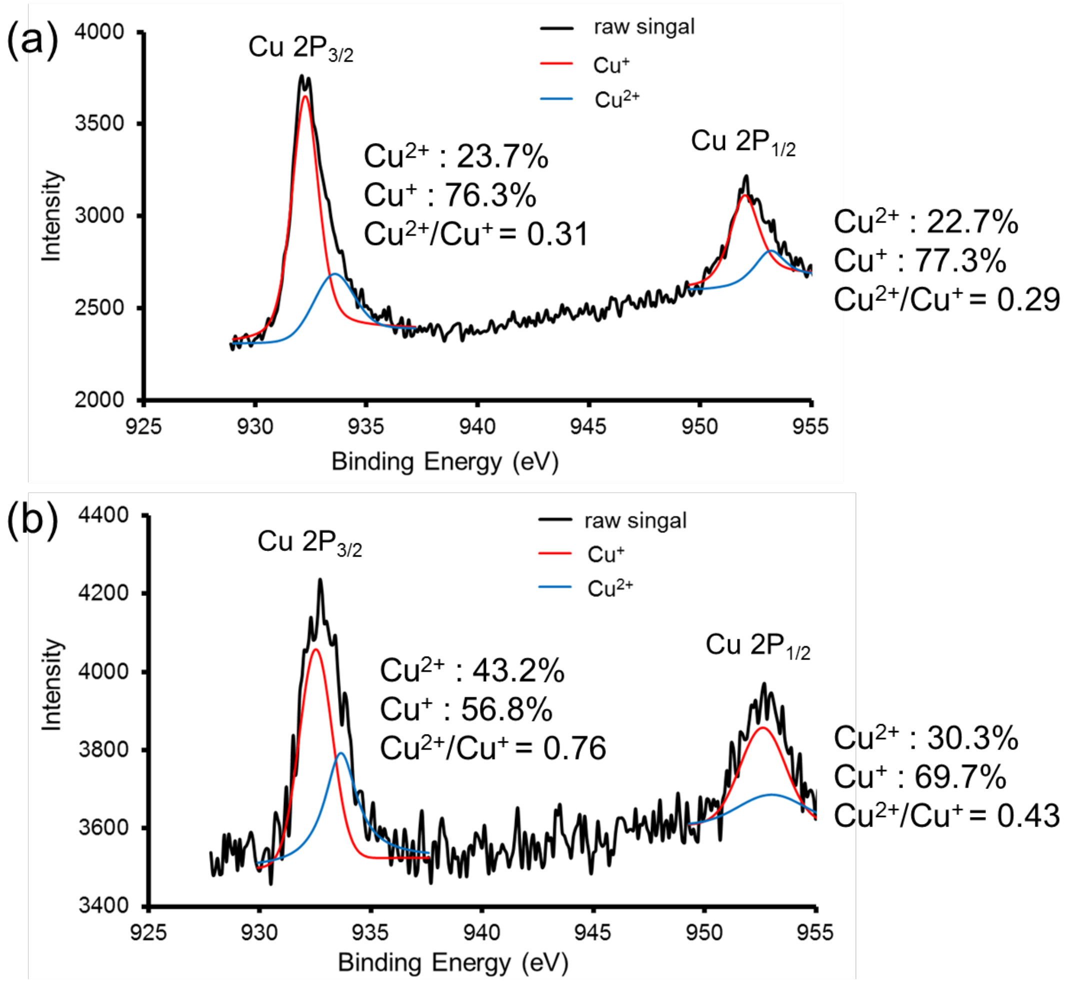

Figure S5. XPS analysis of GSNO/$Cu_{1.6}S$-PLGA polymersomes before and after irradiation with 633 nm laser at 1 W/cm^2 for 15 min. (a) Before irradiation, the binding energies of the GSNO/$Cu_{1.6}S$-PLGA polymersomes showing peaks at 932.6 ($2P_{3/2}$) and 952.5 ($2P_{1/2}$) eV assigned to Cu^+ and the peaks at 933.6 ($2P_{3/2}$) and 954.1 ($2P_{1/2}$) eV attributed to Cu^{2+}. The ratios of Cu^{2+}/Cu^+ calculated as 0.31 at Cu $2P_{3/2}$ and 0.29 at Cu $2P_{1/2}$. **(b)** After irradiation, Cu^{2+} increase with the ratios of Cu^{2+}/Cu^+ as 0.76 at Cu $2P_{3/2}$ peak and 0.43 at Cu $2P_{1/2}$ peak. The XPs signals were deconvoluted to Cu^+ and Cu^{2+}.

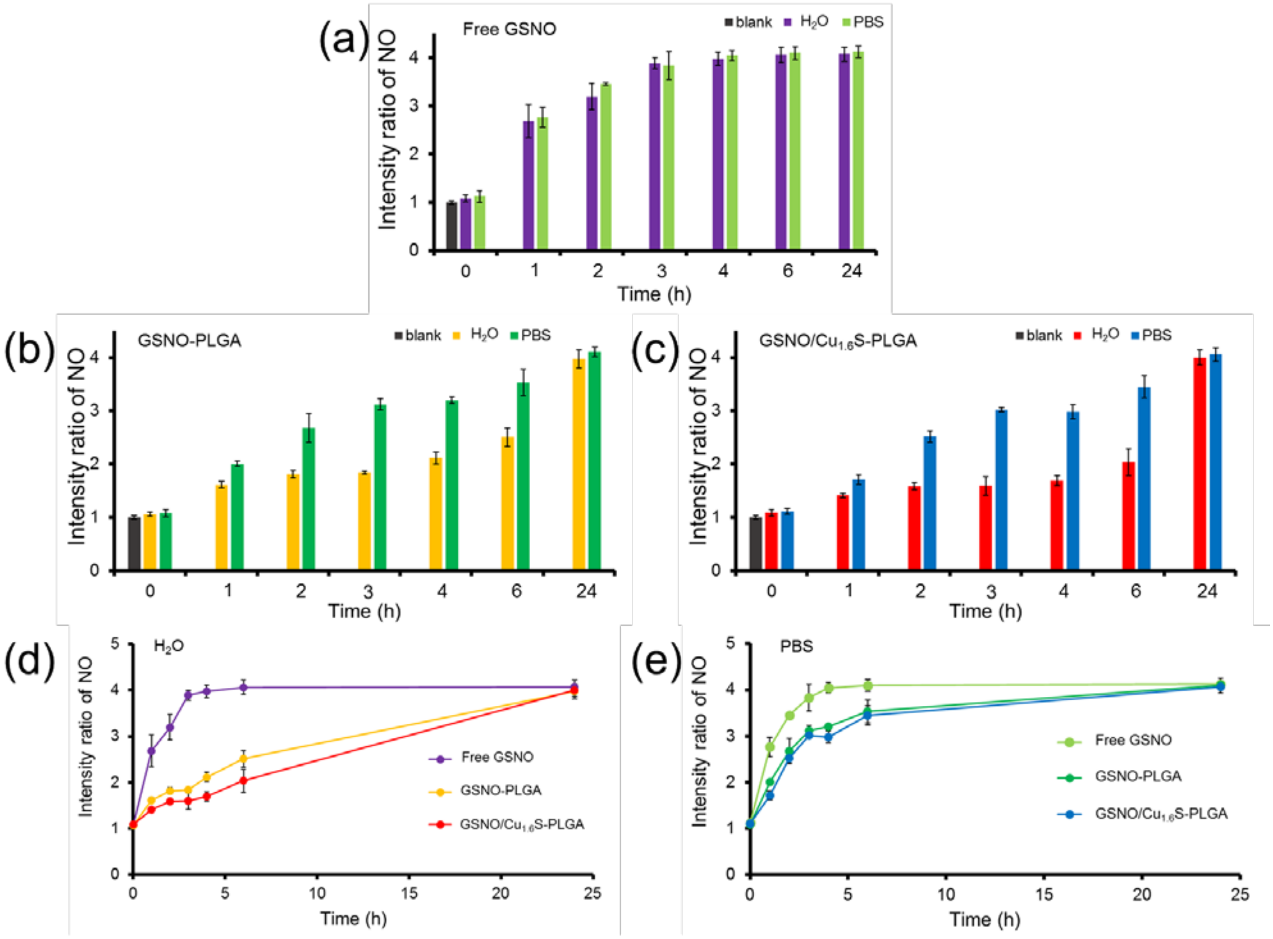

Figure S6. The stability of free GSNO, GSNO-PLGA polymersomes and GSNO/$Cu_{1.6}$S-PLGA polymersomes at 37 °C. (a) The stability of free GSNO (4.12 μM) in H_2O and PBS (pH 7.4). The stability of **(b)** GSNO-PLGA polymersomes and **(c)** 200 ppm (Cu ion concentration) of GSNO/$Cu_{1.6}$S-PLGA in H_2O and PBS (pH 7.4) and the corresponding NO release profiles in H_2O **(d)** and PBS **(e)**. 4.12 μM of GSNO was encapsulated from a 200 ppm (copper ion concentration) of GSNO/$Cu_{1.6}$S-PLGA polymersomes. The intensity ratios of NO release were calculated relative to the blank group. The blank experiments were performed using NO assay kit without addition of polymersomes.

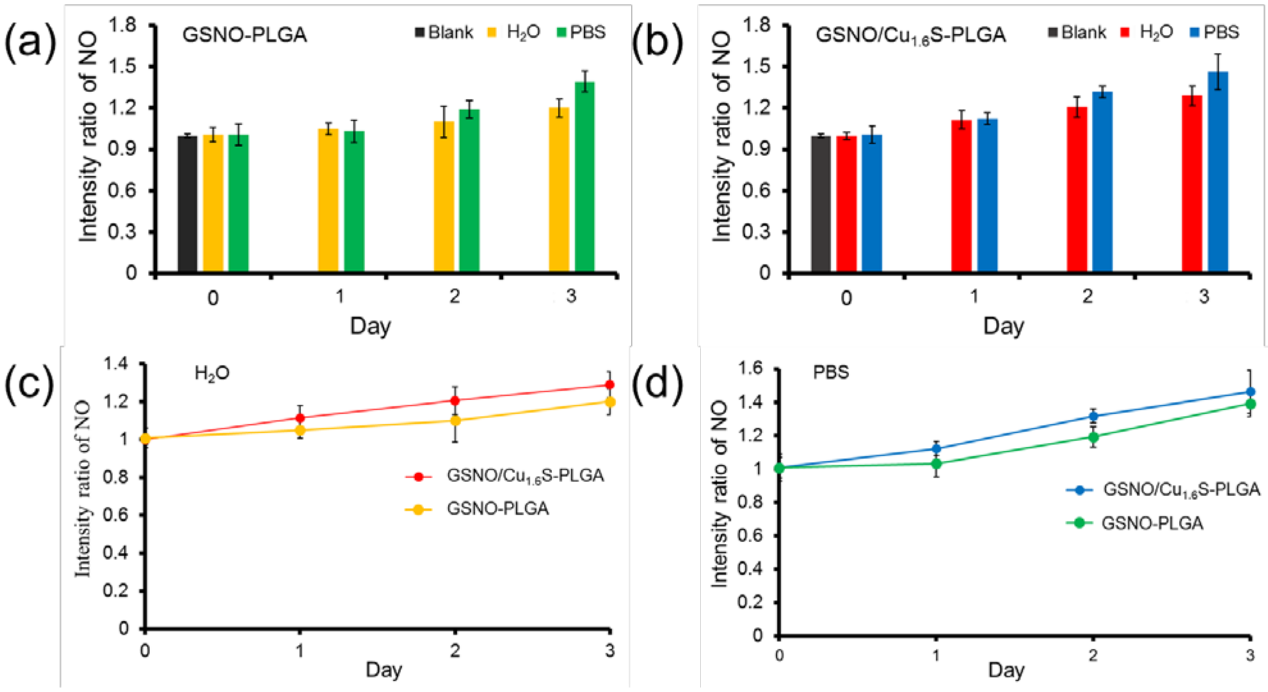

Figure S7. The stability of GSNO-PLGA polymersomes and GSNO/$Cu_{1.6}S$-PLGA polymersomes at 4 °C. The stability of **(a)** GSNO-PLGA polymersomes and **(b)** 200 ppm (Cu ion concentration) of GSNO/$Cu_{1.6}S$-PLGA polymersomes in H_2O and PBS (pH 7.4) and the corresponding NO release profiles in H_2O **(c)** and PBS **(d)**. 4.12 μM of GSNO was encapsulated from a 200 ppm (copper ion concentration) of GSNO/$Cu_{1.6}S$-PLGA polymersomes. The intensity ratios of NO release were calculated relative to the blank group. The blank experiments were performed using NO assay kit without addition of polymersomes.

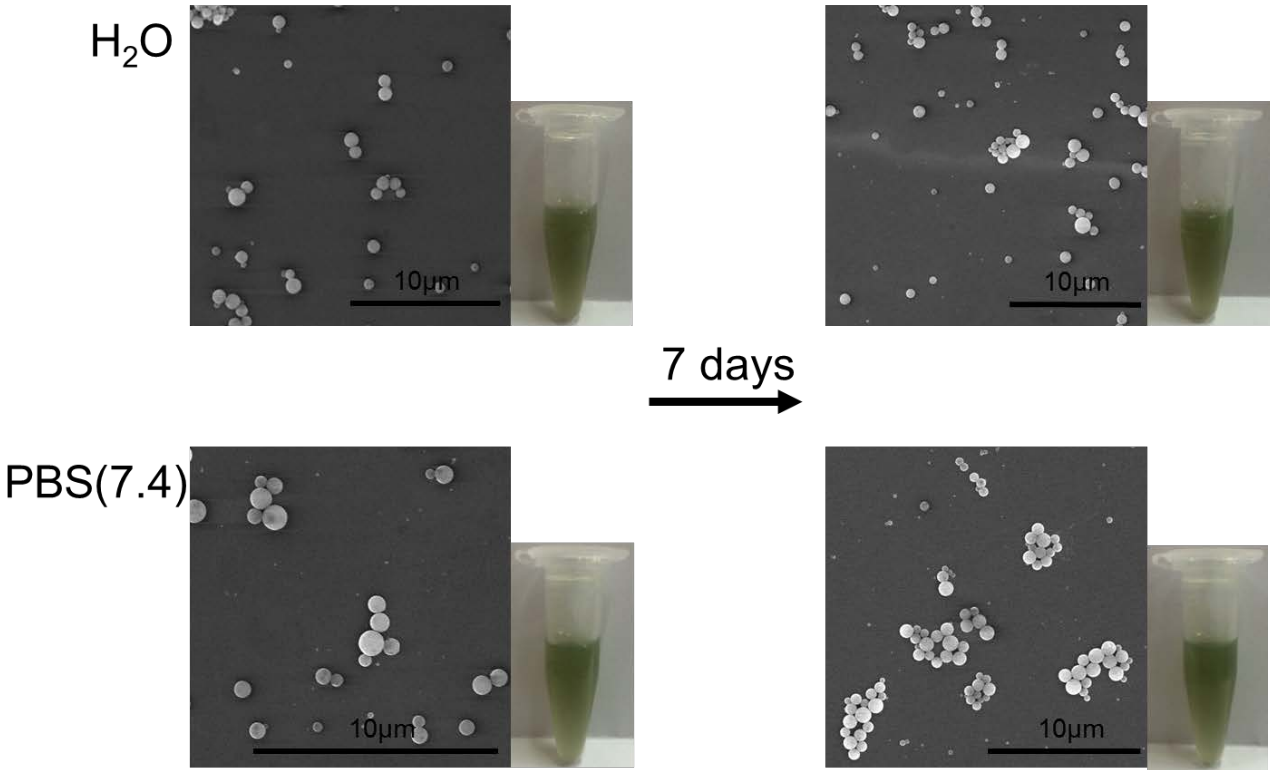

Figure S8. TEM images and the photographs of the polymersome solutions. The GSNO/$Cu_{1.6}S$-PLGA polymersomes were dispersed in H_2O and PBS (pH 7.4) at 4 ^oC and monitored for 7 days. The morphology of GSNO/$Cu_{1.6}S$-PLGA polymersomes was captured by SEM.

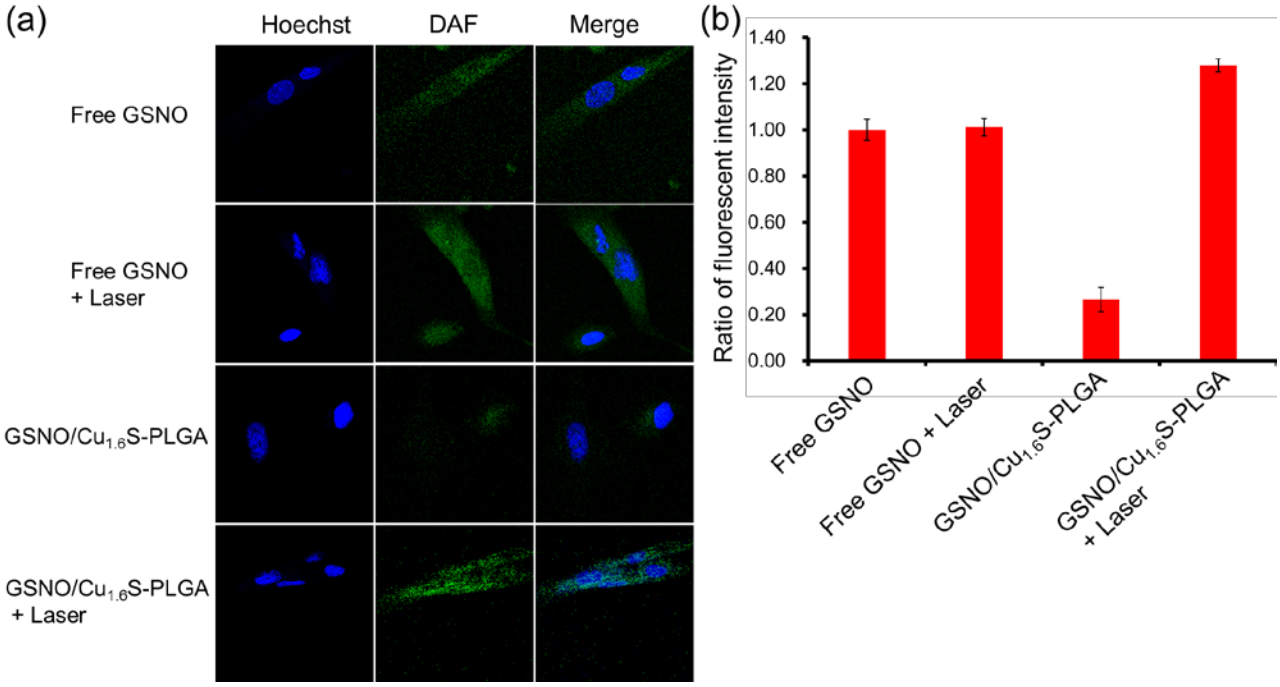

Figure S9. The evidence of the intracellular NO release upon laser irradiation under 3 h incubation. (a) The confocal images of the MRC-5 cells captured under different conditions for free GSNO (4.12 μM) and GSNO/$Cu_{1.6}$S-PLGA polymersomes (200 ppm in Cu ion concentration with total 4.12 μM encapsulation of GSNO) with or without laser exposure. The nuclei were stained with Hoechst (blue) and the green fluorescence was derived from DAF-2 DA. Laser illumination was performed for 15 min exposure using 633 nm laser at 1 W/cm^2. **(b)** The fluorescent intensities corresponding to **(a)** figure were calculated relative to the free GSNO group. The cells were incubated with free GSNO or GSNO/$Cu_{1.6}$S-PLGA polymersomes for 3 h.

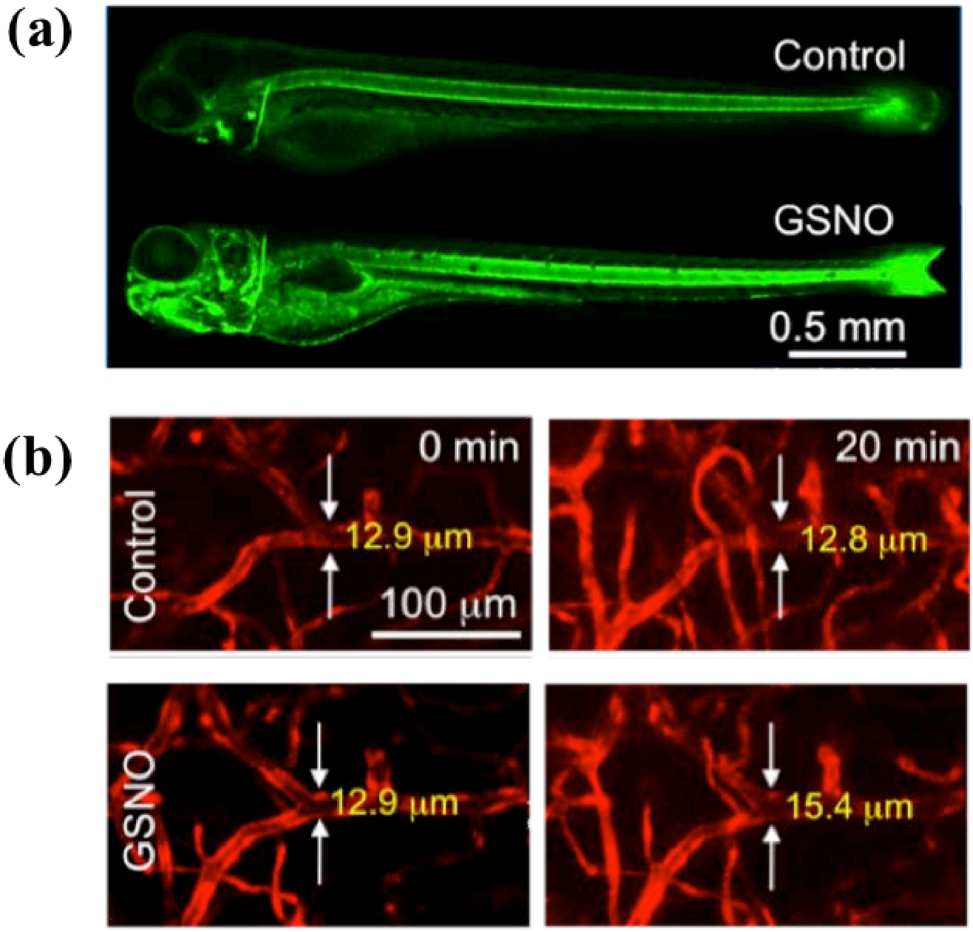

Figure S10. Validation of the protocols used to determine the exogenous generation of NO in living zebrafish and its vasodilatory activity on the cerebral blood vessel of zebrafish. (a) Fluorescence images show that zebrafish (wild type, 5 dpf), which were soaked sequentially with a solution of GSNO (50 μM, 1 h) and a solution of DAF-FA DA (5 μM, 2h) in the dark at 28 °C, exhibited a greater fluorescence in comparison with the control prepared on zebrafish soaked with a solution containing DAF-FA DA alone. The result indicates the possibility of using the fluorescent sensor of NO, DAF-FA DA, to determine the exogenously generated NO in living zebrafish. **(b)** Fluorescence images show a profoundly increased width of the basilar artery for zebrafish (transgenic line, Tg(kdrl:mcherry)) soaked with a solution of GSNO (5 μM, 1 h) relative to the control prepared on zebrafish soaked with a solution containing no GSNO.
